# Preparation and Mechanical Properties of Photo-Crosslinked Fish Gelatin/Imogolite Nanofiber Composite Hydrogel

**DOI:** 10.3390/ma5122573

**Published:** 2012-11-29

**Authors:** Naozumi Teramoto, Akihiko Hayashi, Kaori Yamanaka, Asako Sakiyama, Asuka Nakano, Mitsuhiro Shibata

**Affiliations:** Department of Life and Environmental Sciences, Faculty of Engineering, Chiba Institute of Technology, 2-17-1 Tsudanuma, Narashino, Chiba 275-0016, Japan; E-Mails: akihiko.h06232@gmail.com (A.H.); kaori.y05232@gmail.com (K.Y.); asako.s04231@gmail.com (A.S.); asuka.n03231@gmail.com (A.N.); shibata@sky.it-chiba.ac.jp (M.S.)

**Keywords:** gelatin, fish scale, imogolite, nanofibers, hydrogel, photopolymerization, composite gel

## Abstract

Fish gelatin (FG) extracted from sea bream scales was reacted with glycidyl methacrylate (GMA), and the product (FG-GMA) was used for photopolymerization using a radical photoinitiator in the presence or absence of imogolite nanofibers in the aqueous solution. The synthesis of FG-GMA was confirmed by ^1^H NMR spectroscopy, and photopolymerization of FG-GMA was achieved successfully by irradiation with ultraviolet (UV) light for 3 min to yield translucent composite hydrogels. The concentration of FG-GMA varied from 10% to 30% without imogolite, and that of imogolite varied from 0% to 2.0%. A microtomed gel sample was observed with a transmission electron microscope (TEM), and imogolite nanofibers were found to be dispersed finely in the gelatin matrix. Scanning electron microscope (SEM) observation of the lyophilized gel revealed that it had a porous morphology. Mechanical properties of hydrogels were measured by compression tests using a mechanical tester, and viscoelastic properties were measured using a rheometer. The mechanical strength and storage modulus of the hydrogel increased with an increase of imogolite.

## 1. Introduction

Collagen is the main protein constructing connective tissues in vertebrate animals. Collagen is also a well-known biocompatible material and often used as an extracellular matrix (ECM) for regenerative tissue engineering [1,2,3,4]. Gelatin is denatured and partially degraded collagen extracted by acidic, alkaline and/or hydrothermal processes; and it is also a candidate for ECM used in regenerative tissue engineering [5,6,7,8]. Usually, gelatin is extracted from the skin of domestic mammals, such as pigs, calves and cows. However, gelatin extracted from domestic mammals has a risk of spreading infectious diseases, including bovine spongiform encephalopathy (BSE). Marin gelatin, represented by fish gelatin, is an alternative to mammalian gelatin and expected to lower this risk [9,10,11]. The largest advantage of marine gelatin is that it is inexpensively available by extraction from seafood processing wastes. Gelatin and collagen are the major polymer products from skin, scales and bones of fishes in the seafood processing industry.

Collagen has a triple-stranded helix structure, and the structure partially remains in gelatin. The partial triple-stranded helix structure in many fish gelatins, however, is denatured in the aqueous solution at room temperature, because the denaturing temperature of fish collagen is lower [12]. This means that such fish gelatin is not gelling, but soluble in water at room temperature. The denaturation, as well as partial degradation, causes the weakness in mechanical properties of fish gelatin, and it has limited its application fields. Some research groups reported the reinforcement of gelatin materials [13,14,15,16,17,18,19,20,21,22,23,24,25]. To achieve the reinforcement of gelatin materials, they have often adopted strategies of crosslinking and/or compositing with other materials. Yadav *et al.* reported preparation of gelatin gel assembled with hydroxyapatite reinforced by multiwalled carbon nanotubes for artificial bone grafting applications [26]. 

Recently, several high-performance hydrogels, a double network gel [27], a topological gel [28], a nanocomposite gel [29] and a tetra-PEG gel [30], have been developed. Among these, the technique for the nanocomposite gel can be easily applied to various gels. We were inspired by the nanocomposite gel, and decided to prepare nanofiber-reinforced fish gelatin gel using imogolite as nanofibers. Imogolite is often seen in the clay fraction of soils derived from glassy volcanic ashes [31]. It is a hydrated aluminosilicate polymer with a tubular structure: An external diameter of *ca.* 2 nm, an internal diameter of *ca.* 1 nm and a length from several hundred nanometers to several micrometers. We have already investigated the interaction between imogolite and type I collagen from calf skin and found that collagen was closely bound to imogolite through the electrostatic interaction at pH = 4 to pH = 8 [32]. Therefore, gelatin has a great chance of interacting with imogolite. While collagen from calf skin precipitates in the presence of imogolite, fish gelatin does not precipitate in the presence of imogolite. Most recently, Shikinaka *et al.* reported on imogolite-reinforced acrylamide gel [33]. They found a great reinforcing effect for imogolite. Reinforcement of polymer materials with imogolite was also reported by Takahara’s group [34,35,36]. Liu *et al.* used halloysite, another clay nanotube, to prepare the nanocomposite hydrogel with high equilibrium degree of swelling [37]. 

**Scheme 1:**
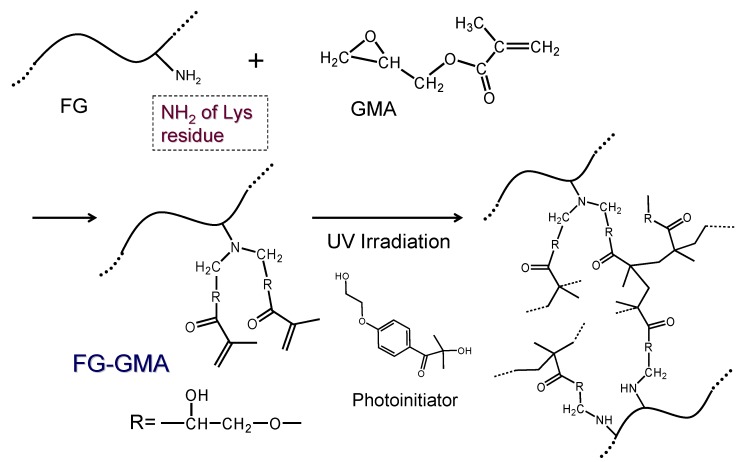
Preparation and photopolymerization of fish gelatin (FG) modified with GMA.

Collagen and gelatin from mammalian sources are less soluble in neutral water at room temperature and easily become hydrogel. On the other hand, fish gelatin is soluble in water, and we should formulate a strategy for gelation of fish gelatin. Chiou *et al.* reported crosslinking of cold water fish gelatins by glutaraldehyde and genipin [16]. Kim and Uyama reported enzymatic crosslinking of cold water fish gelatin using transglutaminase [17]. Bhat and Karpin reported slight crosslinking by ultraviolet light irradiation [15]. In the present study, we formulated a strategy of crosslinking fish gelatin carrying methacrylate groups by UV irradiation in the presence of a radical photoinitiator. Fish gelatin was first reacted with glycidyl methacrylate (GMA), and the pendant methacrylate groups were used for radical photopolymerization (Scheme 1). Considering future applications for making 3D-shaped materials for cell culturing, as an example, photopolymerization is one of the desirable candidates for gelation methods for the purpose of making designed objects from fish gelatin. Photoencapsulation of chondrocytes in poly(ethyleneoxide) gel using methacrylate groups was reported by Elisseeff *et al.* [38]. More recently, Chou *et al.* evaluated photo-crosslinked alginate hydrogel *in vivo* for matrix accumulation by nucleus pulposus cells, and they proved the safety of photopolymerization of methacrylate groups in the presence of vital cells [39]. We examined the effect of the addition of imogolite on the mechanical and rheological properties. 

## 2. Experimental

### 2.1. Materials

Fish gelatin (*M*_n_ 21,000 g/mol and *M*_w_ 29,000 g/mol measured by GPC with light scattering), which was extracted from the scales of red sea bream with hot water, was supplied by Kanemoto Kaisan Co., Ltd. (Chiba, Japan). Aluminum (III) chloride hexahydrate and glycidylmethacrylate (GMA) was purchased from Kanto Chemical Co., Inc. (Tokyo, Japan); and tetrasodium monosilicate n-hydrate (Si ~22%) was purchased from Wako Pure Chemical Industries, Ltd. (Osaka, Japan). 2-Ethyl-4-methylimidazole was purchased from Tokyo Chemical Industry Co., Ltd. (Tokyo, Japan). These chemical reagents were used as received. Irgacure 2959, 2-hydroxy-1-[4-(hydroxyethoxy)phenyl]-2-methyl-1-propanone, was kindly provided from Ciba Specialty Chemicals K. K. (Tokyo, Japan) and used as received. Ultra pure water (electric resistance >18 MΩ∙cm^−1^) used for gel preparation was obtained through Milipore Direct-Q and used for preparation of imogolite and hydrogel.

### 2.2. Synthesis of Methacrylate-Modified Fish Gelatin (FG-GMA)

Fish gelatin from red sea bream (FG) (2.1 g) was dissolved in 20 mL of dimethylsulfoxide (DMSO), and 2-ethyl-4-methylimidazole (0.2 g) was added. After 8.7 mL of GMA was added dropwise to the solution, the solution was stirred at 800 rpm for 24 h at 28 ± 1 °C using EYELA ChemiStation PPS-5510 personal organic synthesizer (Tokyo Rikakikai Co., Ltd.). After the reaction, the solution was poured dropwise into excess ethanol, and the mixture was stirred for 2 h at room temperature. The mixture was filtered with filter paper, and the product on the filter paper was washed with ethanol. To remove the residual volatile impurities, the product was dissolved in 50 mL of ultra pure water and lyophilized for 3 days to yield white powder (FG-GMA, yield 71%).

### 2.3. Synthesis of Imogolite

Imogolite was synthesized by the method developed by Suzuki *et al.* [40] as follows: to prepare the reactant solutions, 3.35 g of tetrasodium monosilicate n-hydrate (Si ~22%) (0.012 mol) was dissolved in 200 mL of water; and 7.24 g of aluminum chloride hexahydrate (0.030 mol) was dissolved in 200 mL of water. To the solution of AlCl_3_ was added 2Na_2_O∙SiO_2_ solution dropwise and stirred for 20 min at room temperature. Then 1 N NaOH was added at a rate of 1 mL/min using a syringe pump under stirring until the pH of the mixture reached 7.0. The mixture was centrifuged at a rate of 3500 rpm for 10 min with polypropylene (PP) centrifuge tubes, and the supernatant was removed to obtain aluminosilicate gel. For the purpose of washing out the excess salt, enough water was added to the PP centrifuge tubes to suspend the product, and the suspension was poured into 400 mL of water in a 500 mL beaker. The suspension was stirred for 1 h at room temperature, and then centrifuged at a rate of 3500 rpm for 10 min, followed by removing the supernatant. This washing procedure was repeated three times. The precipitate, a translucent gel, was suspended in 2 L of water, and the pH of the suspension was adjusted to 4.0 with 0.5 N HCl. After stirring for 2 days at room temperature, the suspension became almost transparent. The suspension was divided into two 1 L PP screw capped bottles and aged for 2 days in an electric oven set at 105 °C. Then the suspension was lyophilized for 2 days to yield a white fibrous powder (yield 25%). The X-ray diffraction (XRD) analysis also confirmed the synthesis of imogolite.

### 2.4. Preparation of Gelatin/Imogolite Composite Hydrogel by Photoirradiation

Synthetic imogolite was dispersed in 50 mM sodium acetate buffer at pH 4.0, then stirred for 24 h. The dispersion was sonicated for 30 s at the power of level 4 (corresponding to 80 W output) and the frequency of 20 kHz using a Branson Sonifier 250 ultrasonic homogenizer (Branson Ultrasonics Co.), and then FG-GMA was dissolved in the imogolite dispersion at the prescribed concentration. The morphology of imogolite nanofibers did not change during the sonication. To the mixture was added Irgacure 2959, a water-soluble radical photoinitiator, at the concentration of 0.1 wt %. The mixed liquid sample was poured into a small Teflon container, and irradiated with UV light (~60 mW/cm^2^) using a Spotcure SP-7 UV irradiator (Ushio Inc.). In preparation of gel samples for compression tests, a Teflon container with an inner diameter of 12 mm and an inner height of 22 mm was used; and in preparation of gel samples for rheology measurement, a Teflon container with an inner diameter of 30 mm and an inner height of 10 mm was used. The height of the sample was 8 mm for the compression test, and 1.7 mm for the rheology measurement. The gel samples for compression tests were used as prepared; and the gel samples for rheology measurement were cut out using a pipe punch with an inner diameter of 20 mm. To remove light of wavelength shorter than 300 nm, the irradiation was carried out through a glass plate of 1 mm thickness.

### 2.5. Characterization

X-ray diffraction (XRD) analysis was performed at ambient temperature on a Rigaku RINT-2100 X-ray diffractometer at a scanning rate of 1.0°/min using Cu Kα radiation (wavelength, λ = 0.154 nm) at 40 kV and 14 mA. Imogolite was analyzed in powder form. Proton nuclear magnetic resonance (^1^H NMR) spectra were recorded on a Bruker AV-400 (400 MHz) using DMSO-*d*_6_ and D_2_O as a solvent.

The surface morphology of lyophilized gel samples was observed by a Hitachi S-4700 field emission scanning electron microscope (FE-SEM); the accelerating voltage was 1 kV or 5 kV; and the samples were coated with gold prior to observation. The nanodispersion of imogolite in the composite gel samples was observed by a Hitachi H-7100FA transmission electron microscope (TEM). The accelerating voltage was 100 kV. The wet hydrogel sample was sectioned using a cryomicrotome, and the resulting ultrathin section was supported on a grid followed by drying in the pre-evacuation chamber of the TEM.

The compression test was carried out by a Shimadzu EZ-S tabletop universal tester with a 100 N load cell at a crosshead speed of 1 mm/min using cylindrical specimens with a diameter of 12 mm and a height of 8 mm. In each case, five specimens were tested and the average values were calculated. The dynamic viscoelasticity of the hydrogel was measured by a DAR-100 rheometer (Rheologica Instruments) equipped with a plate geometry with a diameter of 20 mm. The storage and loss moduli (G' and G'') were determined as a function of frequency under the condition of a linear viscoelastic response at 25 °C.

## 3. Results and Discussion

### 3.1. Synthesis of Methacrylate-Modified Fish Gelatin (FG-GMA)

**Figure 1:**
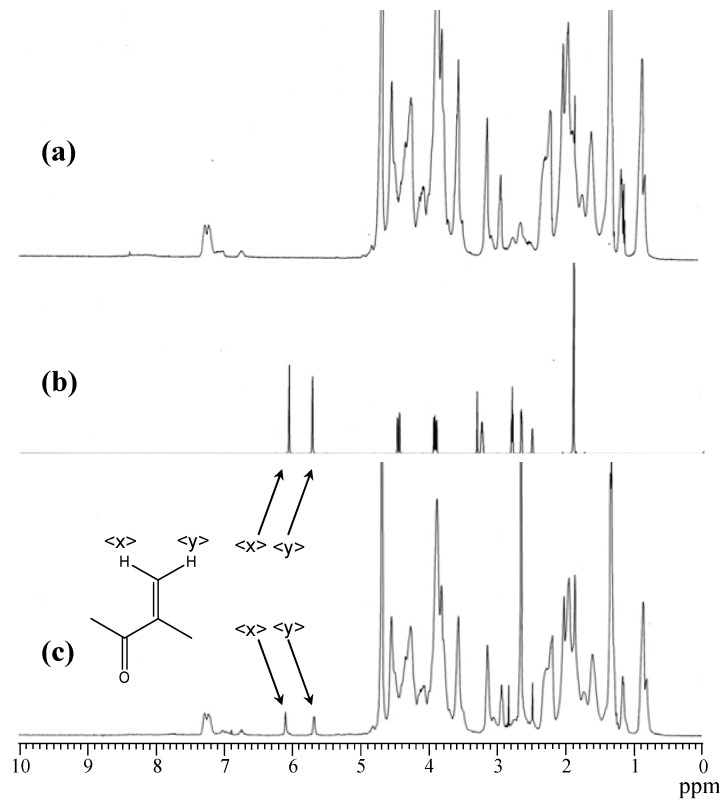
^1^H NMR spectra of (**a**) FG; (**b**) GMA; and (**c**) the product (FG-GMA).

The gelatin we used in the present study was extracted from scales of sea bream, and its molecular weight was lower than that of native fish collagen, because of hydrolysis during extraction with water at high temperature and high pressure. The fish gelatin, which is soluble in water and DMSO at room temperature, was reacted with GMA in DMSO in the presence of a base catalyst. After the purification and lyophilization, the dried white cake, which is soluble in water and DMSO, was obtained. Figure 1 shows the ^1^H NMR spectra of fish gelatin (FG) and the product. Most signals in the spectrum of the product coincide with those of FG, except two signals corresponding to unsaturated methacrylate groups. These two signals were seen at 6.1 ppm and 5.7 ppm. The signal of methyl protons of methacrylate groups was observed at 1.9 ppm. Considering that two GMA molecules generally react with one amino group of a Lys residue present in FG, the extent of the reaction can be calculated from the intensity of the signals and the Lys content in FG (2.83 mol %). The extent of the reaction calculated from ^1^H NMR was 28%. 

### 3.2. Preparation of Gelatin/Imogolite Composite Hydrogel by Photoirradiation

Imogolite synthesized in our laboratory was dispersed only in acidic buffer solution, and we used sodium acetate buffer (pH 4.0) to prepare the hydrogel. FG-GMA was dissolved in the imogolite dispersion at the concentration of 10%, 20% or 30% by weight. The final concentration of imogolite was varied from 0.2% to 2.0% against the whole weight of the hydrogel. Hydrogel samples were designated as “FG X-Imo Y”, where X is concentration of FG-GMA and Y is imogolite content (in weight percentage). Among these samples, we could not prepare the FG30-Imo2.0 hydrogel sample, because the sample solution before UV irradiation became highly viscous and resulted in inhomogeneous gelation.

**Figure 2:**
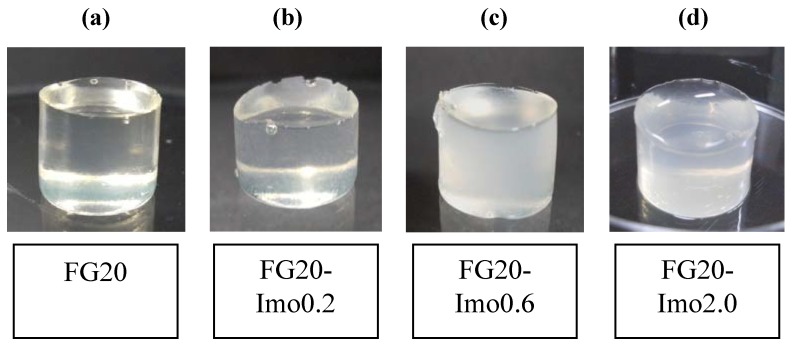
Digital photographs of composite hydrogels of FG and imogolite: (**a**) FG20; (**b**) FG20-Imo0.2; (**c**) FG20-Imo0.6; and (**d**) FG20-Imo2.0.

During photopolymerization, the sample solution was irradiated repeatedly at interval of 15 s irradiation, accompanied by 10 s cooling to prevent the solution from heating. The total irradiation time was 3 min for each sample. After irradiation with UV, a self-standing hydrogel was obtained. In the absence of imogolite, we obtained a semi-transparent hydrogel; and the transparency decreased with an increase of imogolite content (Figure 2). However, the transparency and turbidity did not change when imogolite content was 0.6% and more. The turbidity is due to the difference in the refractive index between imogolite and gelatin. The concentration of gelatin did not affect the transparency.

### 3.3. Electron Microscopic Observation of Lyophylized Gelatin/Imogolite Composite Gel

**Figure 3:**
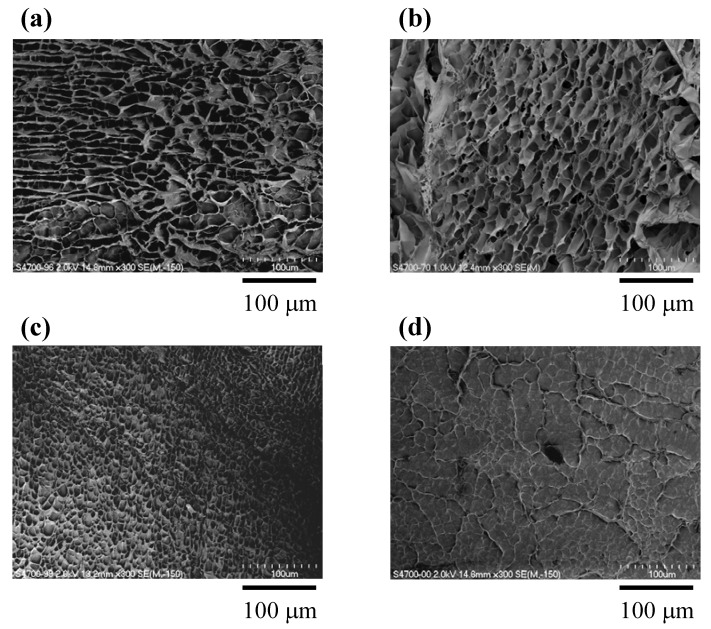
SEM photographs of lyophilized composite gels: (**a**) FG20; (**b**) FG20-Imo0.2; (**c**) FG20-Imo0.6; and (**d**) FG20-Imo2.0.

Surface morphology of the lyophilized gel was observed by FE-SEM and shown in Figure 3. All samples exhibited the porous morphology, and the pore size became smaller as imogolite content increased. The surface morphology of the lyophilized gel sample containing 2.0 wt % imogolite was different from the gel samples with lower imogolite content. Many pores were closed up by a matrixlike a wall, most probably due to deformation of the inner-side pores caused by rapid evacuation of water through small pores during the lyophilization process. 

**Figure 4:**
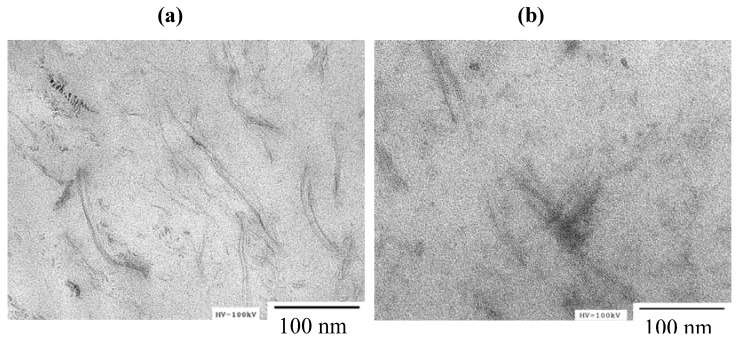
TEM photographs of composite gels sectioned using a cryomicrotome: (**a**) FG20-Imo0.4 and (**b**) FG20-Imo2.0.

The dispersion state of imogolite nanofibers in the gel was observed by TEM. Figure 4 shows TEM photographs of FG20-imo0.4 and FG20-imo2.0. The TEM observation of FG20-imo0.4 revealed that imogolite nanofibers spread homogeneously in the FG matrix; and the result suggests that imogolite did not aggregate, but interact with FG. In our previous study, we reported on the complex products of imogolite with type I collagen from calf skin [32]. According to the previous study, the interaction between imogolite and type I collagen was so strong that precipitation occurred immediately by mixing type I collagen and imogolite, and we came to the conclusion that the major interaction was probably an electrostatic interaction. In the present study, however, no precipitation occurred when we mixed fish gelatin and imogolite. The different behavior is caused by the difference in molecular structures between type I collagen and fish gelatin, as well as the difference in the molecular weight. Type I collagen from mammals maintains a triple helix structure at room temperature, and its solubility is not so high. In contrast, fish gelatin derived from fish collagen is generally denatured at room temperature, resulting in an untwisting of the triple helices of peptide chains. The solubility of fish gelatin is high. Considering that imogolite was finely dispersed in the FG matrix, there exists weak interaction between imogolite and FG. On the other hand, the TEM photograph of FG20-imo2.0 showed fine dispersion and partial aggregation of imogolite. The result suggests that the interaction between imogolite and FG was not enough to disperse imogolite nanofiber at higher concentration.

### 3.4. Physical Properties of Gelatin/Imogolite Composite Hydrogel

**Figure 5:**
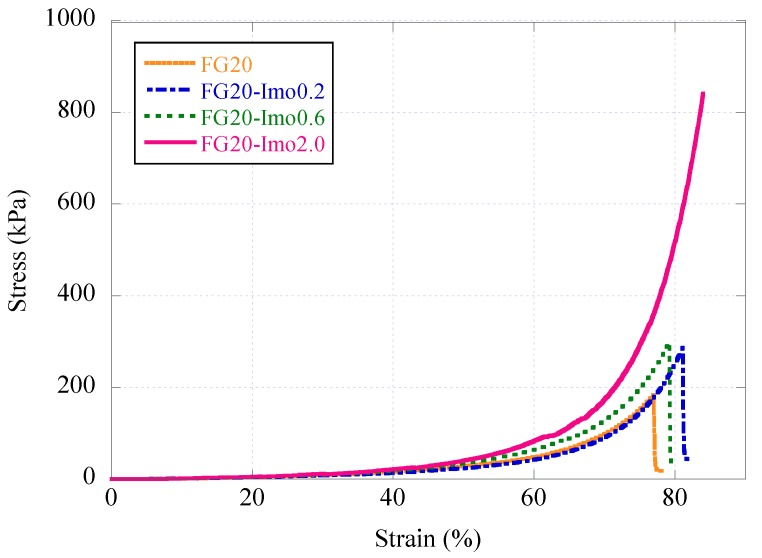
Stress-strain curves of FG20 composite hydrogels in the compression test. Imogolite content was varied from 0% to 2.0%.

Mechanical properties of fish gelatin/imogolite composite hydrogels were measured by compression tests. Figure 5 shows the results of stress-strain curves in the compression tests of FG20 series. The compression stress at break (compression strength) increased by the addition of imogolite, and moderate reinforcement was observed at the imogolite content of 0.2 wt % and 0.6 wt %. In contrast, a significant increase of compression strength was observed at the imogolite content of 2.0 wt %, and the highest compression strength was ~800 kPa. Aggregation of inorganic fillers in polymers generally reduces their improvement effect. In spite of the partial aggregation of imogolite in FG20-imo2.0, the compression strength increased compared with FG20-imo0.6. We suggest that imogolite nanofibers are so fine that they can keep the reinforcing effect, even if they aggregate partially. 

**Figure 6:**
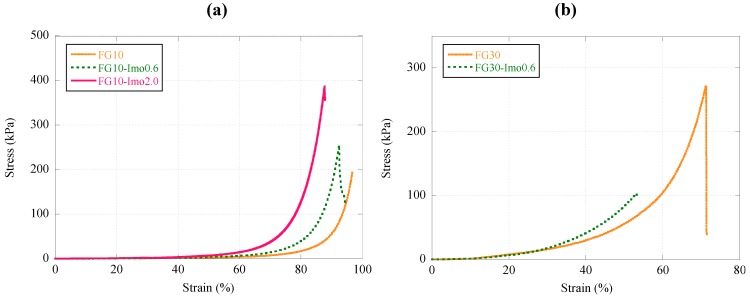
Stress-strain curves of FG10 and FG30 composite hydrogels in the compression test: (**a**) FG10 series and (**b**) FG30 series.

Figure 6a,b shows the results of stress-strain curves in the compression tests of FG10 series and FG30 series, respectively. While we observed moderate improvement of compression strength with imogolite for FG10 series, addition of imogolite diminished the compression strength for FG30 series. The compression strength of FG30-imo0.6 was lower than FG30 without imogolite. FG30-imo0.6 was a very brittle gel, and the strain at break of FG30-imo0.6 became low, resulting in lower toughness.

**Figure 7:**
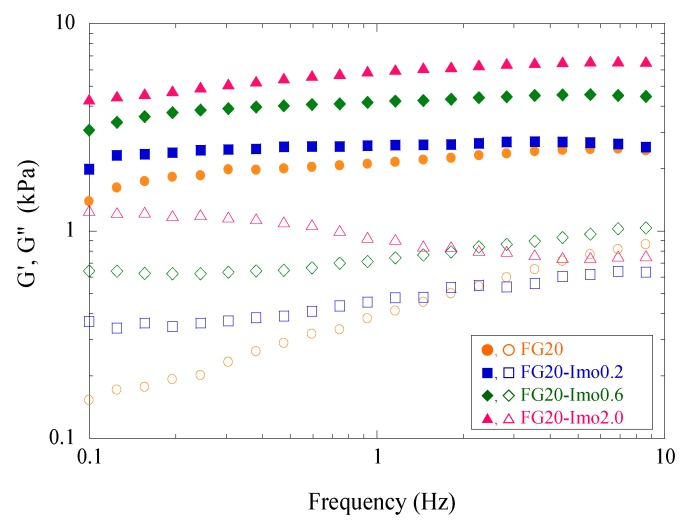
Frequency dependency of storage modulus, G' (filled symbols), and loss modulus, G'' (open symbols), for FG20 composite hydrogels at different imogolite content varied from 0% to 2.0%.

Figure 7 shows the viscoelasticity of fish gelatin/imogolite composite hydrogels in the frequency range of 0.1–10 Hz. All samples show the typical gel behavior, with storage modulus (G') being higher than loss modulus (G'') over the whole frequency range. Since G' relates to elasticity and G'' relates to viscosity, the comparison of G' with G'' is very important for the judgment between viscoelastic fluid and viscoelastic gel [41]. G' of photo-crosslinked samples was independent of oscillatory frequency and increased with an increase of imogolite content. The results indicate the effective reinforcement of fish gelatin gels with imogolite nanofibers. Though G'' somewhat changed with frequency change, G'' did not cross over G' for all samples. Regarding viscoelasticity of FG20, G'' increased gradually, with an increase of frequency getting close to G'. The FG20 gel showed more viscous behavior at the higher frequency region than the lower frequency region, implying the partial existence of a loose network. In contrast, G'' showed higher values at a lower frequency region for FG20-imo2.0. This type of viscoelastic behavior is often observed in the measurement of viscoelasticity of clay suspensions at higher concentration [42,43,44]. As imogolite content becomes higher, a three-dimensional network of imogolite nanofibers gradually emerges. The partial aggregation found in the TEM observation is considered to relate to this result.

## 4. Conclusions

We proposed a novel approach for photopolymerization of fish gelatin and reinforcement with imogolite nanofibers. Fish gelatin modified with methacrylate groups was synthesized using glycidyl methacrylate as a modification reagent and was irradiated with UV light in the presence of imogolite and a radical photoinitiator to obtain translucent hydrogels. The morphology of the lyophilized hydrogel was revealed as highly porous by SEM observation. As a result of TEM observation, imogolite was found to be finely dispersed in the composite gel with 0.4% imogolite. In the composite gel with 2.0% imogolite, however, partial aggregation of imogolite was observed. In spite of the aggregation, the mechanical properties of the hydrogel were remarkably improved at the imogolite content of 2.0%, when the concentration of the modified fish gelatin was 20%. The hydrogel samples showed the viscoelastic properties typical of gel, with the storage modulus being higher than the loss modulus over the frequency range from 0.1 Hz to 10 Hz. 
